# Quantitative, Qualitative and Geospatial Methods to Characterize HIV Risk Environments

**DOI:** 10.1371/journal.pone.0155693

**Published:** 2016-05-18

**Authors:** Erin E. Conners, Brooke S. West, Alexis M. Roth, Kristen G. Meckel-Parker, Mei-Po Kwan, Carlos Magis-Rodriguez, Hugo Staines-Orozco, John D. Clapp, Kimberly C. Brouwer

**Affiliations:** 1 Department of Medicine, Division of Global Public Health, University of California San Diego, San Diego, CA, United States of America; 2 Joint Doctoral Program in Public Health, Global Health, University of California San Diego and San Diego State University, San Diego, CA, United States of America; 3 Drexel University School of Public Health, Philadelphia, PA, United States of America; 4 Department of Geography and Geographic Information Science, University of Illinois at Urbana-Champaign, Champaign, IL, United States of America; 5 Centro Nacional para la Prevención y el Control del VIH y el SIDA (CENSIDA), Mexico City, Mexico; 6 Facultad de Medicina, Universidad Autónoma de Ciudad Juárez, Ciudad Juárez, Mexico; 7 Ohio State University, Columbus, OH, United States of America; David Geffen School of Medicine at UCLA, UNITED STATES

## Abstract

Increasingly, ‘place’, including physical and geographical characteristics as well as social meanings, is recognized as an important factor driving individual and community health risks. This is especially true among marginalized populations in low and middle income countries (LMIC), whose environments may also be more difficult to study using traditional methods. In the NIH-funded longitudinal study *Mapa de Salud*, we employed a novel approach to exploring the risk environment of female sex workers (FSWs) in two Mexico/U.S. border cities, Tijuana and Ciudad Juárez. In this paper we describe the development, implementation, and feasibility of a mix of quantitative and qualitative tools used to capture the HIV risk environments of FSWs in an LMIC setting. The methods were: 1) Participatory mapping; 2) Quantitative interviews; 3) Sex work venue field observation; 4) Time-location-activity diaries; 5) In-depth interviews about daily activity spaces. We found that the mixed-methodology outlined was both feasible to implement and acceptable to participants. These methods can generate geospatial data to assess the role of the environment on drug and sexual risk behaviors among high risk populations. Additionally, the adaptation of existing methods for marginalized populations in resource constrained contexts provides new opportunities for informing public health interventions.

## Introduction

Place, in terms of its physical characteristics and social meanings, is recognized as an important influence on individual health risks [1, 2]. In terms of HIV, prevention efforts that have ignored place and only focused on individual behavior have resulted in incomplete risk reduction [3–5]. Rhodes et. al. defines the HIV risk environment as “the space, whether social or physical, in which a variety of factors exogenous to the individual interact to increase vulnerability to HIV” [3]. Interventions aimed at modifying the HIV risk environment represent an opportunity to address both core causes of health disparities and improve individual-level interventions [4].

Low and middle income countries (LMIC) have felt the brunt of the HIV epidemic, yet most of the tools to characterize the HIV risk environment have been designed in high-income settings. Studies suggesting the importance of social disorder or spatial distance in access to HIV care may be context specific, and not applicable outside of high-income settings. Thus, adapting and applying these methods to capture the HIV risk environment in LMIC is sorely needed to address this literature gap.

Female sex workers (FSWs) are at heightened risk for sexually transmitted infections (STIs), including HIV [6]. Because sex work is frequently illegal, the HIV risk environment of FSWs is often relegated to discrete areas (i.e., red light districts) or liminal spaces (e.g., alleyways, underpasses) at the margins of society. Additionally, sex work often intersects with substance use, compounding risk to women who are more likely to work and/or do drugs in isolated spaces, where exposure to violence is greater and access to services is more limited [7–9]. In this paper, we examine the physical and social HIV risk environment of FSWs in an LMIC, including the spatial locations where women work or live, women’s perception of and movement through places (activity space), as well as physical features of these spaces (built environment).

Given the stigmatized nature of sex work and how it is often intertwined with substance use, delineating the HIV risk environment often requires capturing multiple locations (e.g., home, sex work venue, shooting galleries) in order to more fully understand the risk environment [10, 11]. Activity spaces, the places where people undertake their daily activities and traverse during the course of their daily lives [11, 12], provide a more complete look at one’s risk environment. For example, avoidance of areas due to policing has been associated with pressure to have sex without a condom and decreased access to healthcare and syringe exchange programs [13, 14]. Additionally, spatial isolation has been associated with exchanging sex for drugs (a riskier form of exchange than for money) and greater prevalence of HIV and STIs [15, 16].

Within these activity spaces, growing evidence shows that the built environment can both negatively and positively influence substance use and sexual behaviors, which in turn affect HIV/STI risk. The built environment includes “features of human-made spaces, places or surroundings in which human activity takes place” [15]. For instance, dilapidation of the internal or external built environments (e.g., rundown buildings, dirty sidewalks, homes without basic amenities) has been shown to be associated with overdose death, [17], heavy drinking [18], and depression [19]. Conversely, positive aspects of the built environment, such as parks, can increase collective efficacy and improve health outcomes [20].

In built environment studies among FSWs, the unit of analysis is often the venue, which may be classified based on type (e.g., bar vs. street, indoor vs. outdoor) and comparisons are often limited to differences in individual participants who work there, rather than differences in the venue structure itself [13, 21, 22]. A systematic review of sex work venue literature found that categorizing FSWs by venue type alone, without offering details of venue differences, may miss important details in characterizing HIV risk [23].

Social features of environments where FSWs work may influence HIV risk as well [24, 25]. For example, an assessment of indoor venues of FSWs in Canada found that managerial practices, venue safety policies, access to sexual health services/supplies, drug harm reduction, and social cohesion among sex workers were associated with condom use for pregnancy prevention [26].

**Fig 1:**
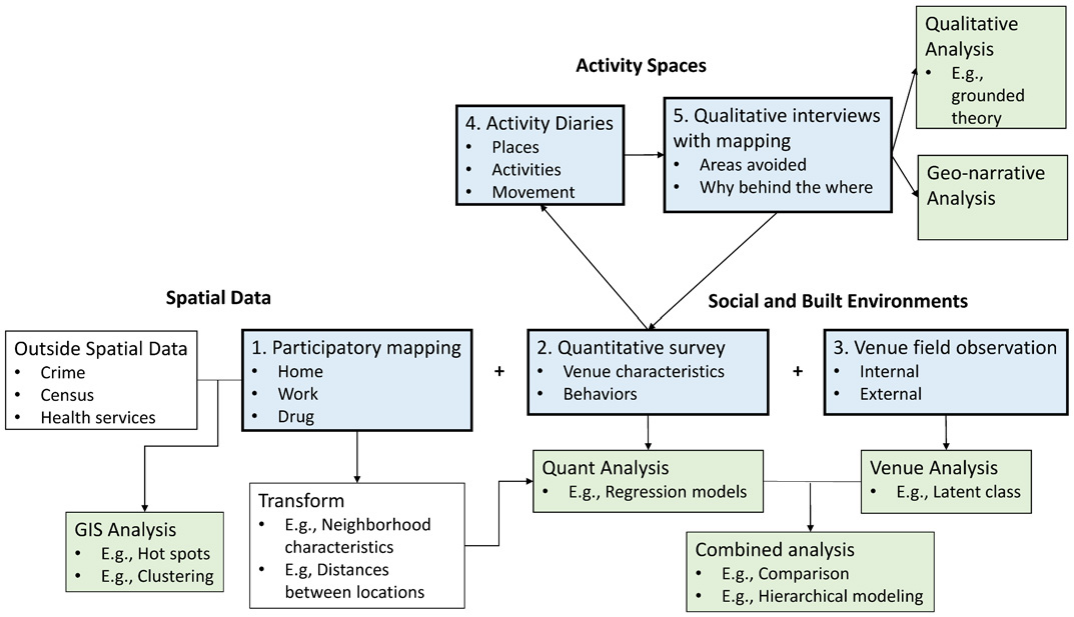
Overview of the five research methods to capture the HIV risk environment and potential analyses. Arrows indicate sequential activities, plus marks indicate concurrent activities.

In this paper we describe the development, implementation, and feasibility of a mix of quantitative and qualitative tools used to capture the HIV risk environments of FSWs in an LMIC setting. The five methods were: 1) Participatory mapping; 2) Quantitative interviews; 3) Sex work venue field observation; 4) Time-location activity diaries; 5) In-depth interviews about daily activity spaces (Fig 1). A secondary aim of this paper was to compare quantitative interview and field observation data in order to evaluate what each method adds to the capture of the HIV risk environment.

We conclude with a discussion of the strengths and weaknesses of these five methods, their applicability to the study of other risk environments, and how the data generated can be integrated to inform interventions aimed at reducing risk. While we use the HIV risk environment of FSWs as a case study, the multi-method approach for capturing spatial, built environment, and activity space data are generalizable to other populations at heightened risk for HIV.

## Setting and Project

The Mexican cities of Tijuana, Baja California and Cd. Juárez, Chihuahua are large metropolitan areas situated along the U.S.-Mexico border. In part due to their location along major northbound drug trafficking routes into the U.S., both cities have heightened levels of substance use, particularly injection drug use [27, 28], as well as established sex trade industries. An estimated 9,000 women work as sex workers in Tijuana and 4,000 work in Cd. Juárez [29]. Substance use and the sex work economies have converged to contribute to a rise in HIV prevalence among high-risk groups. FSWs in these cities have an HIV prevalence of 6% [30], 30 times that of the national prevalence in Mexico [31, 32], while FSWs who also inject drugs have a prevalence of 12% [33].

**Fig 2:**
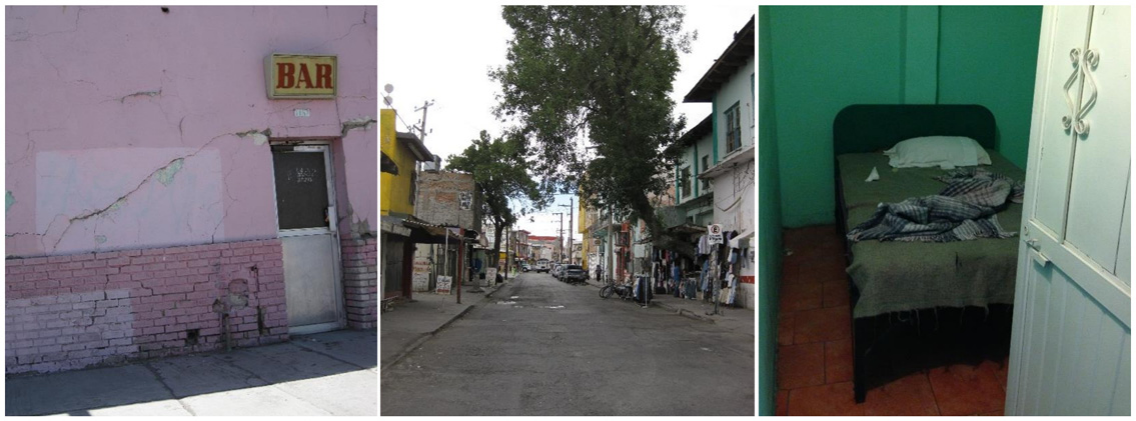
Examples of internal and external sex work venue environments (photo credit Kristen Meckel-Parker).

In both cities, FSWs work in a wide variety of venues including bars, hotels, the street, private homes, and massage parlors (Fig 2). For the purposes of this paper, sex work “venues” are considered to be any place women reported soliciting clients or exchanging sex for money or goods and includes both indoor (e.g., bar, massage parlor) and outdoor (e.g., street) spaces.

*Mapa de Salud*, was a U.S. National Institutes of Health- funded (R01DA028692) longitudinal cohort study of 602 active FSWs recruited from sex work venues in Tijuana and Cd. Juárez from March 2013 to March 2014. Details on study design and eligibility are described in detail elsewhere [34]. Briefly, inclusion criteria included being at least 18 years of age, biologically female, reporting having exchanged sex for money or goods in the past month, agreeing to treatment for any STIs detected, providing written informed consent, and residing in Tijuana or Cd. Juárez with no plans to move out of these cities within 18 months from the time of recruitment. Enrolled women were followed for up to 18 months. All study activities were approved by the Institutional Review Boards of the University of California, San Diego; El Colegio de la Frontera Norte in Tijuana; and the Universidad Autónoma de Cd. Juárez.

All five methods were collected during the approximately two-year study period. Quantitative interviews were conducted at six month intervals. Participatory mapping occurred both during the quantitative interview visits and every three months at “check-in” visits. The sex work venue field observation began after the start of the quantitative interviews and continued on an ongoing basis as new venues were added. Finally, participants were recruited for the activity diaries and qualitative interviews based on characteristics measured in the quantitative interview.

## 1. Participatory Mapping

LMIC can pose challenges to capturing location data [10, 35]. For instance, neighborhood or census level data may be unavailable and block grids or addresses may be less structured. Further, many urban settings in LMIC are undergoing rapid expansion, leaving structural details on new neighborhoods at the periphery of cities less defined. In Tijuana and Cd. Juárez, street addresses are often inconsistent (e.g., numbering is not sequential), but the use of participatory mapping has previously been found to improve response rates in this type of setting [4]. We improved upon the participatory mapping methods by using electronic rather than paper maps, as well as taking advantage of Google Street View.

During baseline and subsequent follow-up visits every three months, participants were asked to provide the locations of where they live, conduct sex work, and use drugs (if applicable). Collecting locations over multiple visits allowed us to capture both locations in space and movement over time. Using Google Maps and Street View as a visual, interviewers worked alongside participants to identify locations. Most participants could identify the *colonia* [neighborhood] in which the activity took place, which assisted in focusing the search. The participant and interviewer then worked together to narrow down locations in the neighborhood through discussion of landmarks, cross-streets, major roads, etc. Once a location was found, the interviewer moved from map view to street view to verify that the correct location was identified and a temporary pin was placed on the map in order to determine the latitude and longitude of the location. When participants did not report having a fixed location (e.g., they walked different streets to solicit clients or used drugs at a variety of private homes), but were confined to a *colonia*, the location was assigned to the *colonia*-level. All coordinates were captured in Google Maps, recorded in an Excel spreadsheet, and imported into ArcGIS (ESRI corp., Redlands, CA, USA). Data were then combined with digital maps of *colonias*, census tracts (AGEBs), roads, and other infrastructure from the Mexican National Institute of Statistics, Geography, and Informatics Instituto Nacional de Estadística Geográfica Informática (INEGI) or other municipal sources.

Despite the collection of sensitive information, no participants declined to provide details on the requested locations. Field staff reported that participants enjoyed the interactive aspect of data collection and seeing familiar locations in satellite and street view. The most common barrier to locating a point was outdated satellite imagery on Google Maps, especially in cases of newly developed areas of the city. In those cases, the interviewer would work with the participant to select a point in the approximate area based on geographic context and note the issue in the Excel spreadsheet.

## 2. Quantitative Interviews

At 6 month intervals, participants were given an interviewer administered quantitative survey using computer-assisted personal interviewing (CAPI). This survey included questions on sociodemographics, current and past substance use, sexual behaviors and experiences, sex work history, HIV knowledge, community and personal violence experiences, incarceration history, and their work environment. Participants also received laboratory testing for HIV/STIs.

The quantitative interview included a series of questions regarding participant’s perceptions of the physical and social characteristics of their primary sex work venue and surrounding neighborhood. Questions included details about physical characteristics (number of other SW who work there, presence of a security gate, pests, lighting, noise level), proximity to other locations (bars, pharmacies, public transportation), availability of condoms or free syringes, police presence, substance use activities, venue policies (e.g., drinking alcohol with patrons) and characteristics of the surrounding neighborhood (e.g., presence of street violence). As with the spatial data collection, women participating in the study openly reported on even very sensitive topics (e.g., substance use, history of violence) during the interview [34, 36].

## 3. Sex Work Venue Field Observation

We adapted physical and built environment evaluation tools to develop a venue checklist measurement tool designed to capture observable sex work venue and block-face characteristics. Items were informed by the Systematic Social Observation Inventory (SSO i)[37] [38], The Urban Built Environment and Overdose Mortality in New York City Neighborhoods[17], Revised Block Environmental Inventory Instrument [39], input from Dr. John Clapp and his natural bar observation [40], and previous research among FSWs in Mexico [41–43]. Existing checklists informed items capturing social disorder [39], physical disorder [38, 39], and internal venue environment [17, 40]. However, because none of the checklists had been conducted in LMIC, nor were there any standardized questions relevant to sex work at the time of development, items were revised to fit the local context.

After we developed a draft of the sex work venue checklist, four researchers used it to independently rate two venues in Tijuana and then debriefed immediately afterwards to discuss the face validity of items. Modifications were made through consensus. For example, items used in other surveys such as “cracked or missing sidewalks” were felt to be too ubiquitous in Tijuana and Cd. Juárez to be useful indicators of neighborhood disorder. The checklist was revised and pilot tested again in the field. Once the checklist was translated into Spanish, it was sent to field team members for a review of face validity and cultural relevance. Final measures in the checklist were categorized into the following domains: general (physical and social) characteristics, policing, drugs/alcohol, safety, physical or social disorder, and public health (S1 Table).

### Definition of block space

Venues were located in a wide variety of areas (e.g., shopping centers, next to major roadways, alleyways, etc.) and were often not on a traditional block-grid system, nor was there clear zoning demarcating industrial vs. business vs. residential areas. As part of the piloting process, researchers designed a standardized method for defining the size of the block space. Decision rules were created for defining non-standard block space situations including: venues on corners, in strip malls, or on unusually wide or long streets (Table 1). These definitions guided the collection of data on the outdoor physical environment of each venue. Following the block space decision rules, polygons were drawn around the points of each primary venue reported by participants. All maps and block polygons were created using Google Maps. Fieldworkers were provided with instructions on these decision rules as well as the final map of block spaces.

**Table 1:** Definitions of exterior space boundaries, “block spaces”, of sex work venues.

Type	Definition of block space
Typical block	Both sides of the street bounded by the intersections
Corner venues	Length of two buildings along the street in all directions (forming a T or cross shape). Empty lots were also included in the building count.
Wide streets	Defined as two or more lanes of traffic, usually with a divider. Block space included only the side of the street with the venue; the opposite side of the street or median were not considered part of the block space
Strip malls	Defined as a group of storefronts with a parking lot in front. Space was bounded by the parking lot and included the other storefronts in the mall. If venues were outside the strip mall, but the strip mall was part of the block space, staff were instructed not to enter the mall to do the checklist.
Long streets	External venue space was considered to be the length of approximately 5 buildings to each side of the venue. If present, alleyways or small intersections were used to bound the external space.

### Venue checklist observer piloting and training

Training of observers for conducting the venue checklist included an hour long PowerPoint presentation that introduced each question on the checklist and included photographs illustrating different situations (e.g., a “dark” venue versus a “bright” venue). The observer training utilized recommendations for reliability by Zenk at al., including photographic examples of questions, practice observations, and a question and answer session to gain feedback [44]. The PowerPoint presentation was followed by a mock checklist of the study office and surrounding block, with training staff on hand to answer questions.

Next, two teams of three observers conducted checklists on the same five venues. Responses of observation teams were compared using Cronbach’s Alpha. No venues were below the pre-determined cutoff of 80% agreement. Individual checklist items were compared by calculating the percent agreement and items at 50% agreement or less were considered very poor. Very poor items were clarified through additional training, revised, or removed.

### Data collection

Checklists were conducted on all primary venues reported by participants at baseline. In order to correspond more closely with peak sex work business hours, data collection times were from 8pm to 12am on Thursdays-Saturdays. "Outdoor" questions about the block space were conducted on all venues, but "indoor" questions were only conducted on indoor venues that allowed access to staff. Generally, observers were barred from entering hotel rooms and private homes. Observers used a computer tablet to record venue checklist data. If observers felt unsafe carrying the tablet, they had the option to complete the checklist on a paper form and enter data into the tablet later. The checklist took an average of 25 minutes to complete.

At each site, two trained observers experienced with recruitment and interviewing participants evaluated all of the venues at baseline. The decision to use pairs was for safety reasons. To avoid unwanted attention or affecting the behavior of staff or patrons within the venues, observers often completed the checklist immediately after leaving the venue, pretended to be clients, or traveled in personal vehicles rather than branded study vehicles.

### Comparing observer checklist to self-report

For this paper we compared project data generated from participant self-report to that from the venue checklist, in order to evaluate the strengths and weaknesses of each approach in capturing HIV risk environments. Items that were the same across the self-report and checklist were recoded into the same response categories if needed. Percentage agreement (PA) between responses were calculated for each item and a pre-determined cutoff of 75% was selected for moderate to high agreement. If there was sufficient variability in each of the ratings, Cohen’s kappa statistic was calculated. All analyses were run using SPSS 23.

We collected data in a staggered manner, with venue data being collected in Tijuana first. Of the 301 participants in Tijuana who completed quantitative surveys, 260 worked at venues accessible to observation. Among these women, 60 reported a primary venue type (e.g., bar, street) in the quantitative survey that differed from the type given during participatory mapping, and were excluded from the analysis.

In comparison to participant self-report, more “underground” characteristics of indoor venues such as the use of drugs (0% vs. 671%, kappa = N/A, PA = 22%), sale of drugs (0% vs. 24%, kappa = N/A, PA = 0%), or availability of condoms (4% vs. 37%, kappa = 0.09, PA = 15%) were less likely to be captured by the venue checklist. In contrast there was fair agreement between the observer checklist and self-report for physical characteristics of venues including the presence of a cover charge (0% vs. 30%, kappa = N/A, PA = 97%), security guard at the door (70% vs. 60%, kappa = 0.5, PA = 81%), bright lighting (12% vs. 23%, kappa = 0.3, PA = 78%), dancing (66% vs. 60%, kappa = 0.3, PA = 75%), and number of sex workers in venue [10 or less vs. more than 10] (57% vs 65%, kappa = 0.5, PA = 77%). Observers were more likely than participants to report feeling safe in venues (100% vs. 60%, kappa = N/A, PA = 60%), with no reports of feeling a little or very unsafe.

## 4. Time-Location Activity Diaries

In other studies, activity spaces of the HIV risk environment have been defined by calculating distances between daily activities [11] or by creating buffers around locations of risk, such as around sex work venues or red light districts [15, 16]. While these traditional geographic information system (GIS) methods are useful for making calculations of distance and assessing the interrelationship between features in the environment, meanings of distance are subjective and GIS fails to capture the social contexts affecting people while they move through space [35, 45]. Coupling ethnographic or narrative analysis with GIS has been developed as a technique to understand both spatial and social contexts of activity spaces [46, 47]. Therefore, a subset of 34 participants in the *Mapa de Salud* study were asked to keep a time-location activity diary over the course of a 7-day period. Building upon methods from Kwan et al, these daily diaries were used to capture the geographical and temporal characteristics of participant’s daily activities, including those unrelated to sex work [47, 48]. Participants were asked to record the time, place, main activity conducted at that place (e.g., slept, ate, used drugs, worked, played with their kids), mode of transportation, and how they felt (e.g., happy, neutral, angry) for places visited throughout the day.

**Fig 3:**
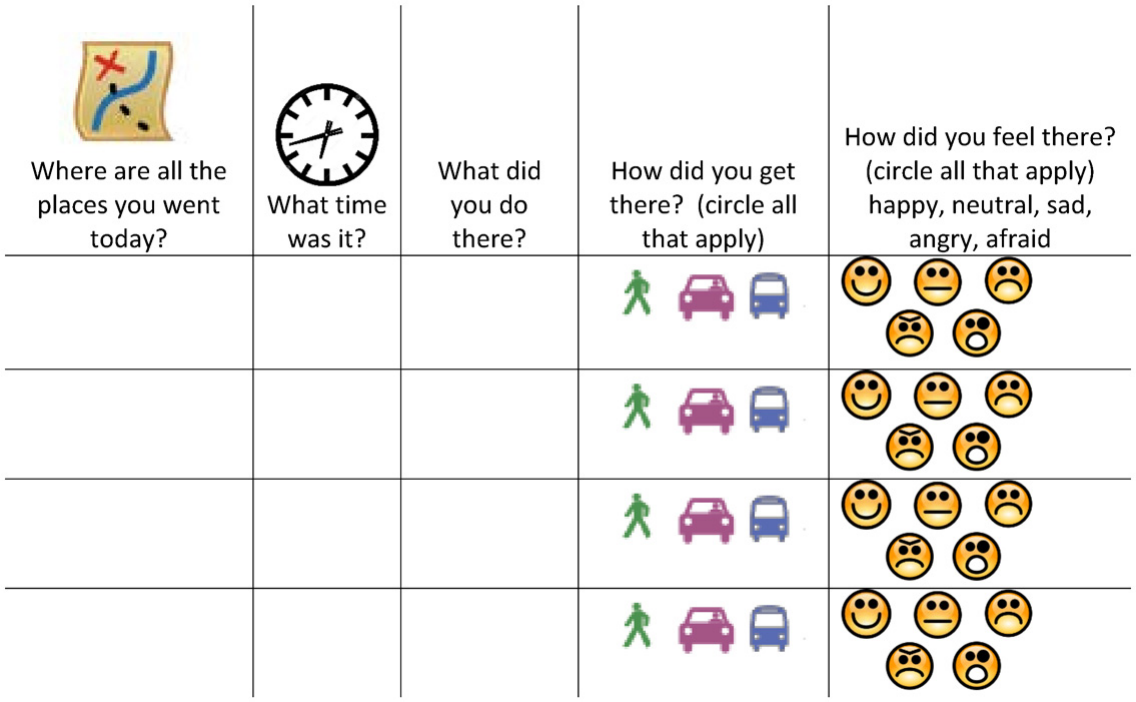
English-version format of a daily activity diary page developed for use in low literacy populations.

Because the median years of education for sample participants was 8 (less than the mandatory level of “secundaria” education in Mexico) [34], the diary included icons that participants could circle to indicate modes of transportation and feelings (Fig 3). This was to facilitate the use for women of all literacy levels. Participants were encouraged to use symbols or codes (e.g., a star) to indicate any illegal activities and pseudonyms for people mentioned. Since they would be expected to carry the diary as much as possible, the first few participants were given choices between different diary sizes. A composition-sized notebook was the preferred size.

All of the women returned for their follow-up interview and all but two brought back the completed diary (one diary was confiscated by police, the other was lost). Daily activities recorded by participants were converted into an excel sheet where each activity was given a unique code number that indicated the participant, day, and time of activity.

## 5. In-Depth Interviews on Activity Spaces

After a week completing activity diaries, participants returned to the study office and debriefed with the interviewer. During this visit they also underwent participatory mapping of all locations traveled to for one typical day of the week. The women then participated in a qualitative in-depth interview about the contextual elements of their work and living environments that may have affected HIV/STI risk. The activity diaries were used as memory tools to help the participants recreate a “day in the life” of their past week.

The semi-structured interviews probed for information about spaces the participant frequented and those they avoided and therefore was able to collect rich information on the mobility of participants and possible constraints on mobility. The type of data collected from these types of interviews can be seen from this excerpt:

*Well*, *I feel safer inside of a hotel*, *you know*? *If anything ever happens to me*, *the person in charge will check on the rooms and if anything happens the person next door will call the police*, *right*? *In fact*, *when I worked on the streets no one could have helped me*, *right*? *That’s the reason why*, *well… that’s why I feel more comfortable there*.

*(Participant*, *age 35*, *Ciudad Juárez)*

## Discussion

The combination of longitudinal mapping techniques, qualitative, and quantitative methods allowed us to collect data for the exploration of social, spatial, and physical factors which may have an influence on sexual and drug use risks.

### Spatial data

The mapping of illicit activities can be challenging due to participant unwillingness to report undesirable behaviors and difficulty locating non-traditional venues (e.g., alleyways)- challenges which are compounded in settings without traditional block systems or consistent use of address numbering. To address these difficulties our methodology employed participatory mapping using Google Maps. A benefit of engaging participants in mapping is that it can lead to more trust and empowerment in a typically marginalized group [49]. This trust building is particularly important when trying to elicit locations of stigmatized or illegal behaviors. In Mexico, even among a low-literacy population, names of *colonias* were widely known by participants and were a useful starting point for narrowing down locations. Because addresses were not widely known or even listed on buildings, the use of Google Street View allowed for the identification of specific places. Limitations of using Google Maps were that it was not always current in areas undergoing rapid development (i.e., suburbs) and required access to the internet.

Google Maps is freely available and most field staff were basically familiar with it prior to the research project. Maps could be generated without the use of expensive, sophisticated software, staff training time was minimal, and data collection and analysis was streamlined through the use of electronic sources. This made it an ideal tool for LMIC settings where funds may not be available for mapping tools. While our study focused on the HIV risk environment, these methods are broadly applicable to the mapping of many locations in LMIC.

### Built environment

Information on sex work venues was captured through both an observational checklist and through self-report by women via a quantitative survey. While repeated retesting of reliability for observational checklists is the gold standard, it is not always feasible. We found that training a small team of individuals to conduct all of the observational surveys was one way to reduce reliability concerns. We used block space definitions and maps to create our own standardized data collection areas, even within areas with non-traditional grid spaces.

The results of the comparison of the data captured by observers using venue checklists and participant self-reported venue features highlighted interesting differences between the two methods. “Underground” characteristics (e.g., illegal drug use) were less likely to be captured by the venue checklist. Also, observer-reported perceptions of venues were generally more positive as compared to reports by FSWs. For physical characteristics, these differences were largely eliminated and there was high agreement between the checklist and self-report. Our findings were similar to those by Zenk et al., who found that items that were stable over time, had clear operational definitions and were large and easy to see were more reliable than items that required judgement of quality or quantity [44]. A limitation of our comparison was that there were some inconsistencies in the “primary” workplace reported by women in the spatial visit compared to the quantitative survey. These differences are likely caused by the fact that many participants worked in multiple venues and asking them to choose a “primary” workplace was an artificial distinction. Also, due to an error in the skip pattern of the original survey, we were missing quantitative data for a number of participants, which reduced our sample size for these items.

While repeated measures may be an appropriate gold standard for capturing dynamic aspects of neighborhoods, self-report from participants may be a valid and less resource intensive alternative. Although checklists are seen as a more objective measure of physical attributes of spaces, social characteristics and perceived attributes of venues, such as the perception of how unsafe it is, may be better captured by the individuals whose risk is being assessed. Self-report has the added benefit of being able to capture data on private spaces, such as homes or hotel rooms, that observers did not have access to. Similar to our study, the *Safer Indoor Work Environmental Scale* published by Duff et. al. has previously been used to capture social, policy and physical features of indoor sex work venues in Canada. The findings from their study showed features of the interior work environment of FSW were associated with improved condom use [26] and is the latest study in a growing body of research showing that sex work venues are a critical intervention point [23].

Our venue checklist, developed for a LMIC setting, captured both indoor and outdoor built environments of a wide range of venue types (i.e., the street, bars, hotels, massage parlors). Tests for associations between physical attributes of venues and surrounding neighborhoods and HIV prevalence and risk behaviors can help identify targets for future interventions or outreach. Methods used to study the venue environment may be generalizable to other HIV risk environments, such as shooting galleries or neighborhoods with high HIV prevalence.

### Activity spaces

Finally, the use of activity diaries and qualitative in-depth interviews was a way to provide context to the quantitative and geospatial data collected by the project. Activity diaries allow for the articulation of how a person’s feelings may change when encountering different features in the environment (e.g., negative feelings about a park where she was arrested) and enables one to map not only the places a person frequents, but also those she avoids. Women in the *Mapa de Salud* study found keeping a daily diary, even of illicit activities, to be acceptable. All the participating women returned for their follow-up interview and 94% brought back the completed diary—despite differing literacy levels. The use of symbols and codes was a way to safely allow for the capture of illicit activities. Data from the activity diaries and accompanying interviews can help elucidate barriers to accessing care (e.g., needle exchange, condoms) and inform the placement of health services.

### Integrating methods

Using nomenclature from Creswell, Fig 1. illustrates the main ways in which the data streams were integrated and provides examples of the types of analyses that can be conducted with each method [50]. Quantitative data was used to identify participants for the activity diaries and in-depth interviews. In turn, themes that were uncovered in the qualitative interviews informed the development of questions for later waves of the quantitative survey. Spatial data points and venue observation data were integrated with the quantitative survey during the data analysis phase.

Depending on the specific research question about the HIV risk environment, differing weight can be given to each of the methods when mixing them. For example, built environment characteristics from the venue field observation can be associated with individual health outcomes reported by women in the quantitative survey (e.g., substance use). Spatial distance measures between locations of interest (e.g., home and the red light district) may be explained by women’s experience of place. These examples highlight some of the many research questions one is able to answer about the HIV risk environment using mixed-methods.

### Conclusions

Overall, multi-method approaches are better equipped to capture a more complete picture of the HIV risk environment than any one method alone. The quantitative, qualitative, and geospatial methods used by the *Mapa de Salud* project outlined here were successful in generating data that are being used to assess the role of the risk environment on drug and sexual risk behaviors among FSW within an LMIC. The methods described can generate place-based data used to assess the role of the environment on drug and sexual risk behaviors among high-risk populations in resource constrained settings, but may have broader utility informing health interventions among other marginalized populations.

## Supporting Information

S1 Dataset Minimal dataset for comparing venue checklist to participant reported venue characteristics. (SAV)

S1 Table Observational checklist questions for assessing indoor and outdoor sex work venue environments. (DOCX)
