# Supplementary material for: Quantitative, Qualitative and Geospatial Methods to Characterize HIV Risk Environments
Source: PLoS One. 2016 May 18;11(5):e0155693. doi: 10.1371/journal.pone.0155693 (PMC4871522; doi:10.1371/journal.pone.0155693)
Supplement: S1 Table — (DOCX) [file pone.0155693.s002.docx]

**S1 Table. Observational checklist questions for assessing indoor and outdoor sex work venue environments**

| **INDOOR** |
| --- |
| Type(s) of venue: *street, bar, brothel, hotel, massage parlor, dance hall, restaurant, billiard hall* |
| **General characteristics** |
| Choose all applicable descriptions: *one floor, multi-level, open floor plan, private rooms/partitions (do not count bathrooms, closets, or areas off-limits to patrons)* |
| Choose all applicable descriptions: Juke box, Live music or DJ, Billiards/games, bar, poles (for dancing), dance floor, gambling machines |
| How crowded is it in the venue: *crowded; neutral; few people* |
| Choose all applicable descriptions: *Dancing, nude dancing, private dances, hand jobs, oral sex, sex* |
| Are there nanas* visible? |
| Approximately how many sex workers are there in the venue: <10; 11-25; 26-50; >51 |
| [Are there sex workers] under the age of 18 |
| [Are there sex workers] over the age of 50 |
| Races of clients: *Mexican, white, black, Asian* |
| What is the predominant race of clients: *Mexican, white, black, Asian* |
| Select all the types of clients there are: *<18, Young (18-24 years), middle aged (25-50 years), older (over 50)* |
| What is the predominant age of clients: *<18; 18-24 years; 25-50 years; > 50* |
| **Physical or social disorder** |
| Choose all applicable descriptions: *Smells bad; Uncomfortable temperature (too hot or too cold); Dusty or dirty countertops/surfaces; Broken items (tables, chairs, lights, etc.); Sticky or visibly dirty floors; Trash on counters or floors* |
| What is the level of lighting inside: *lots of light; neutral; dark* |
| How loud is the venue inside: *very; neutral; quiet* |
| How smoky is the venue inside: *very; some; little or none* |
| How clean is the bathroom: *clean; neutral; dirty* |
| **Drugs/alcohol** |
| Mark all that are visible: *Sale of alcohol; Alcohol consumption; Intoxicated people; Use of drugs; Sale of drugs; Drug paraphernalia visible* |
| **Safety or Policing** |
| Mark all that are visible: *Cover charge; Security guard at the door; Security guard in the venue; Police/federales in the venue* |
| **Public health** |
| Condoms available or for sale? |
| Clean/new syringes for sale or available? |
| **OUTDOOR** |
| **General characteristics** |
| Select all that are visible: Bars/clubs/s*ex work venues; Commercial businesses; Personal residences; Churches/schools; Industrial/warehouses; Vacant lot/open space; Parks/playgrounds; Hotels/rooms for rent; Medical clinic/hospital; Pharmacy* |
| Approximately how many sex workers are there on the street: *<10; 11-25; 26-50; >51* |
| In what language are the signs on the street: *Spanish; English; Other (specify)* |
| What is the predominant language of signs on the Street: *Spanish; English; Other* |
| What is the language of the signs outside the venue: *Spanish; English; Other (specify)* |
| What is the predominant language of the signs on the venue: *Spanish; English; Other* |
| How many lanes are there: *one; two; >three* |
| Is there a sidewalk on at least one side of the street? |
| Is the street paved? |
| How busy is the street: *very; neutral; quiet* |
| **Physical or social disorder** |
| Does the venue have any external wall problems (missing bricks, siding, or outside wall material, sloping or bulging outside walls, or major cracks in outside walls)? |
| Are there any units that appear to be vacant? |
| In general, how would you rate the condition of most of the units on the block: v*ery good; moderate; very bad* |
| Is there strewn garbage, litter, broken glass, clothes, or papers on the block face in the street/sidewalk/or public spaces: *a lot; moderate; little/none* |
| Is there spray paint graffiti on the buildings, signs or walls: *very; a little; no graffiti visible* |
| What is the level of lighting on the street: *Bright; Neutral; Dark* |
| What is the level of lighting directly outside the venue: *Bright; Neutral; Dark* |
| On the street there are (check all that apply): *Kids; people sleeping on the street; people yelling (angry, drunken, threatening); private taxis parked or driving by; abandoned cars* |
| **Drugs and alcohol** |
| On the street there are (check all that apply): *people* *obviously intoxicated/under the influence* |
| **Safety or Policing** |
| If you shouted for help, would someone hear you? |
| Are there police/federales on the street? |
| Are there police cars/trucks parked or driving by? |
| Are there public transportation routes (e.g., bus or fixed route taxi) |
| **INDOOR AND OUTDOOR** |
| **Safety** |
| How safe/unsafe do you feel: *very secure, a little secure, neutral, a little unsafe, very unsafe* |
| How un/comfortable do you feel: *very comfortable, a little comfortable, neutral, a little uncomfortable, very uncomfortable* |
| Do you feel observed? |
| Is the atmosphere tense? |
| Is there the threat of violence? |
| *Nanas are women that oversee/manage FSW in venues |
